# A comparison of owner perceived and measured body condition, feeding and exercise in sport and pet dogs

**DOI:** 10.3389/fvets.2023.1211996

**Published:** 2023-10-12

**Authors:** Heidi A. Kluess, Rebecca L. Jones

**Affiliations:** School of Kinesiology, Auburn University, Auburn, AL, United States

**Keywords:** percent fat, kilocalories, sport, pet, treats, walking

## Abstract

Dog obesity is a significant problem in the US and elsewhere. The purpose was to evaluate factors contributing to pet obesity in sport and pet dog owners. Owners were recruited over social media to answer a questionnaire regarding demographics, health, body condition, feeding, exercise and dog related expenses. Owners identified as pet or sport dog owners. We asked owners to measure the pelvic circumference and hock to stifle length in their dogs in order to calculate percent fat. Owners reported that their dogs were in “ideal” body condition. However, percent fat calculated from owner measurements was significantly different between groups (Sport: 16 ± 10%fat; Pet: 24 ± 10% fat; *p* < 0.05) and revealed that over 50% of the dogs were over fat. Owners reported feeding dogs a range of 413 to 1,133 Kilocalories (Kcal) per day that correlated well with dog size (*R* = 0.58; *p* < 0.05). The size of the treats fed was smaller in the Sport dogs (treat was pinky nail to thumbnail sized) than in Pet dogs (treat was bigger than thumb to larger than palm). Owners reported walking their dogs on leash every day for 15–45 min per session. Overall, owners did a poor job in identifying correct body condition of their dogs. This is concerning because 50% of the dogs were over fat. Better understanding of correct body condition and feeding for the level of physical activity is still a critical issue in controlling obesity in pet dogs.

## Introduction

Research in the US, Australia and Europe estimates that 40–60% of pet dogs are obese resulting in lifelong health problems including arthritis, diabetes, poor quality of life and reduced longevity (1, 2). A number of factors likely contribute to this problem, including owner’s knowledge of correct body condition, over-feeding meals and treats and lack of regular exercise (3, 4). Previous work from this lab suggested that owner knowledge of correct body condition was a significant barrier to intervening in the dog’s body condition with food and exercise (4). In the current study we wished to see if we could improve owners’ identification of their dog’s body condition using several strategies including categories (ideal, overweight, etc), a Purina 9 point scale with descriptions, but without the category labels and having the person measure their own dog. We also investigated feeding, exercise and expenditures related to dog ownership. This study focused specifically on dogs that participate regularly in canine sports compared to pet dogs that do not participate in sports. Dogs that compete in canine sports have a high level of physical activity during competitions and presumably when practicing for the event. Canine Sports can include companion sports where the dog and person perform the sport activity together; examples include obedience, rally obedience, agility, Internationale Prufungs-Ordung (IPO) and tracking. Performance sports involve the person, but the dog does most of the physical activity in frisbee sports, scentwork, field trials, herding, lure coursing, and dock diving. Participation in canine sports is increasing in popularity with the American Kennel Club reporting 3 million entries in 22,000 events last year. We hypothesized that sports dog owners would more correctly estimate body condition, sports dogs would have more appropriate feeding regimes, Sports dogs would exercise more than pet dogs and spend more on their dogs.

## Materials and methods

This study was approved by the Auburn University Institutional Review Board and the IACUC. Owners were recruited over social media to answer a Qualtrics questionnaire that included the Dogs and Walking Survey (DAWGS) (5) that was open from February 2021 to Dec 2021. The current study is part of a larger set of data. Owners self-identified as pet owners with no sports participation, previous sport owners or current sport dog owners.

### Dog demographics and health

Dog demographics included the age, relative size, breed, and sterilization status. We asked about previous diagnoses for temporary or permanent health conditions including orthopedic problems, cardiorespiratory problems, and metabolic disease. We also asked about the number of times they visited the veterinarian in the past year.

### Dog body condition

**Figure 1:**
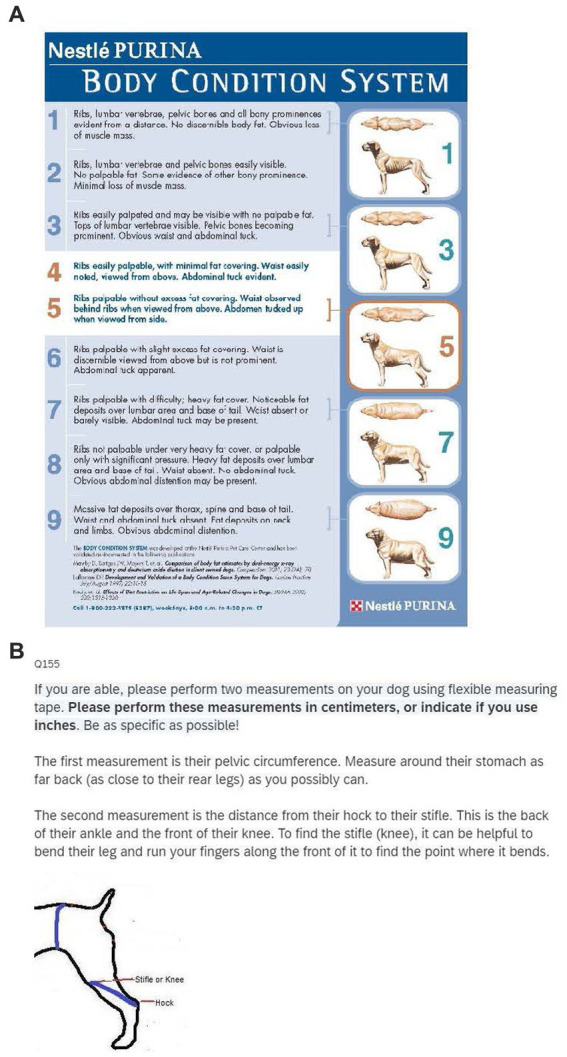
Questions for owners regarding the body condition of their dog. **(A)**. The Purina 9-point scale with the categories removed. **(B)**. Instructions and diagram for owner measurements of their dog.

We asked owners to evaluate body condition using a scale that described visual cues to determine body condition (Purina Body Condition Scale). The scale usually includes labels for each of the categories (“too thin,” “ideal,” and “too heavy”) but we removed those so that owners would use the descriptions and images of the scale and not the labels to judge their dog’s body condition. We also asked them how they would qualitatively rate their dogs body condition (underweight, ideal, overweight, obese). To quantitate body condition, we asked owners to measure the pelvic circumference and hock to stifle length in their dogs (see Figure 1). We used this data to calculate percent fat using the following equations (6):


$$\begin{array}{c} \mathrm{Males}\%\mathrm{fat}=-1.4^{*}\left(\mathrm{hock}\;to\;\mathrm{stifle}\;\mathrm{lengthcm}\right)\\ \;+0.77^{*}\left(\mathrm{Pelvic}\;\mathrm{Circumference}\right)+4. \end{array}$$

$$\begin{array}{c} \mathrm{Females}\%\mathrm{fat}=-1.7^{*}\left(\mathrm{hock}\;to\;\mathrm{stifle}\;\mathrm{lengthcm}\right)\\ \;+0.93^{*}\left(\mathrm{Pelvic}\;\mathrm{circumference}\right)+5. \end{array}$$

The anthropometric measurements for percent fat are validated against DEXA and the Purina Body condition scale.

### Dog feeding

For this section, we asked owners about the type of food (dry, canned, raw, combination, etc.), the brand of food, the formula of food, the number of kilocalories per cup, the amount fed per day, and the number of times fed per day. We used this information to calculate the kilocalories per day for the kibble fed dogs. For treats, we asked about the number of treats, the type of treat, the number of treats given when actively training the dog, and the size of the treat (based on a person’s hand; for example: pinky nail size, thumbnail sized, etc).

### Dog exercise

The amount of exercise a dog received was determined using some basic questions and the DAWGS survey (5). The basic questions included how many times a week they exercised the dog, the main type of exercise, the length of time per session, and the types of activities the dog engaged in without the owner. The DAWGS survey questions included questions about the positivity about dog walking and the positivity about the outcomes from dog walking.

### Sport dog exercise

For owners that indicated that they currently participate in sports, we asked additional questions regarding their sports experience and the number of hours per week they spend on sports-specific training, conditioning or foundation work, walking, running or fetch. We also asked how many days per week the dog was involved in sports or physical activity.

### Dog related expenses

We asked about the amount of money spent on dog care like food, treats, toys, training classes/lessons, and equipment like collars, leashes, etc.

### Data analysis

We used G*Power 3.1.9.7 to calculate the sample size for a MANOVA (power = 0.95, effect size f^2^V = 0.0625). We estimated a total sample size of 172 was sufficient for the power required with 4 groups. We ran a GLM MANOVA with dependent variables percent fat, age, sex/sterility status, size of treats and minutes walked per week using IBM SPSS 29.0.0.0. We did a Tukey *post hoc* test when appropriate. All descriptive data were summarized using mean ± standard deviation. For some variables, we ran a Pearson product–moment correlation using GraphPad Prism 9.4.1. We also ran 1-way ANOVAS with a Tukey *post hoc* test when appropriate. The alpha level was set *a priori* at 0.05.

## Results

### Dog demographics

**Figure 2:**
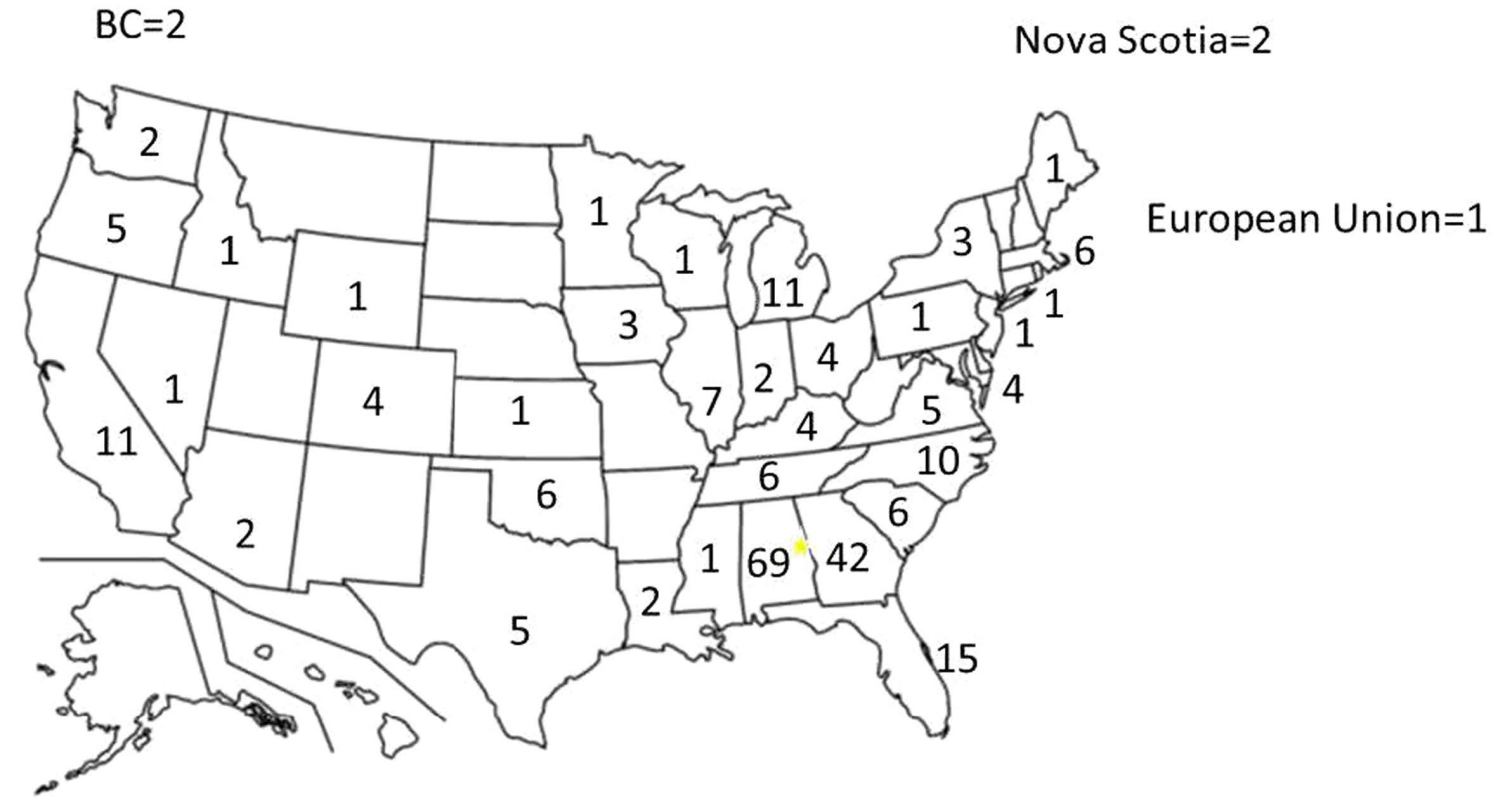
Regional distribution of respondents to the internet survey. The number indicates the number of respondents per state. 33 respondents did not indicate their geographical location.

Overall, 284 self-reported dog owners responded to our online survey. 251 owners responded to the questions regarding location and 246 were from the United States, four from Canada, and one from Europe. Owners from the USA represented 36 states with the largest percent from Alabama, Florida, and Georgia (50%) (see Figure 2 for distribution by state). We asked owners if they were current dog sports participants (Sport), a pet owner (Pet) or a previous sport dog owner (Previous Sport). Participants that did not answer the question were categorized as “Undeclared.” Owners identifying as sport dog owners reported participating in an average of 4 ± 2 dog sports (range: 1–11 sports).

**Figure 3:**
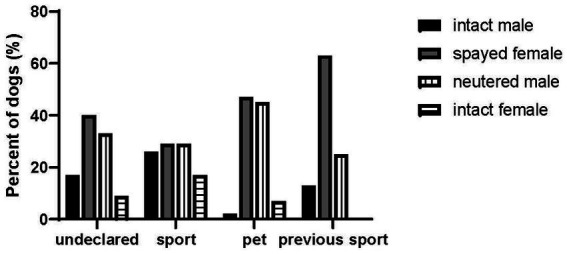
Sterilization status of dog in each group.

The MANOVA results indicated that we had a main effect of percent body fat (*p* < 0.001, *F* = 21.27), age (*p* = 0.007, *F* = 27.98), and size of the treats (*p* = 0.002, *F* = 15.96). The average age of the dogs in the undeclared, sport group and the previous sport group was 5 ± 2 years, but it was 6 ± 3 years for the pet group (*p* = 0.007, *F* = 5.109). The sterilization status of the dogs by group is presented in Figure 3. The Undeclared (74%, 87/120 answered), Pet (91%, 58/58 answered) and Previous Sport owners (88%, 16/16 answered) reported the highest percentage of neutered or spayed dogs. The Sport group (90/90 answered) had the highest percentage of intact dogs (42%). The entire sample represented 68 breeds (including mixed breed as a category). The percent of owner-reported pure breed dogs was 66% (*n* = 87) for Undeclared, 74% (*n* = 90) for Sport, 34% (*n* = 58) for Pet and 10% (*n* = 15) for Previous Sport groups. The owners reported the average size of their dog as medium sized.

### Dog health

**Figure 4:**
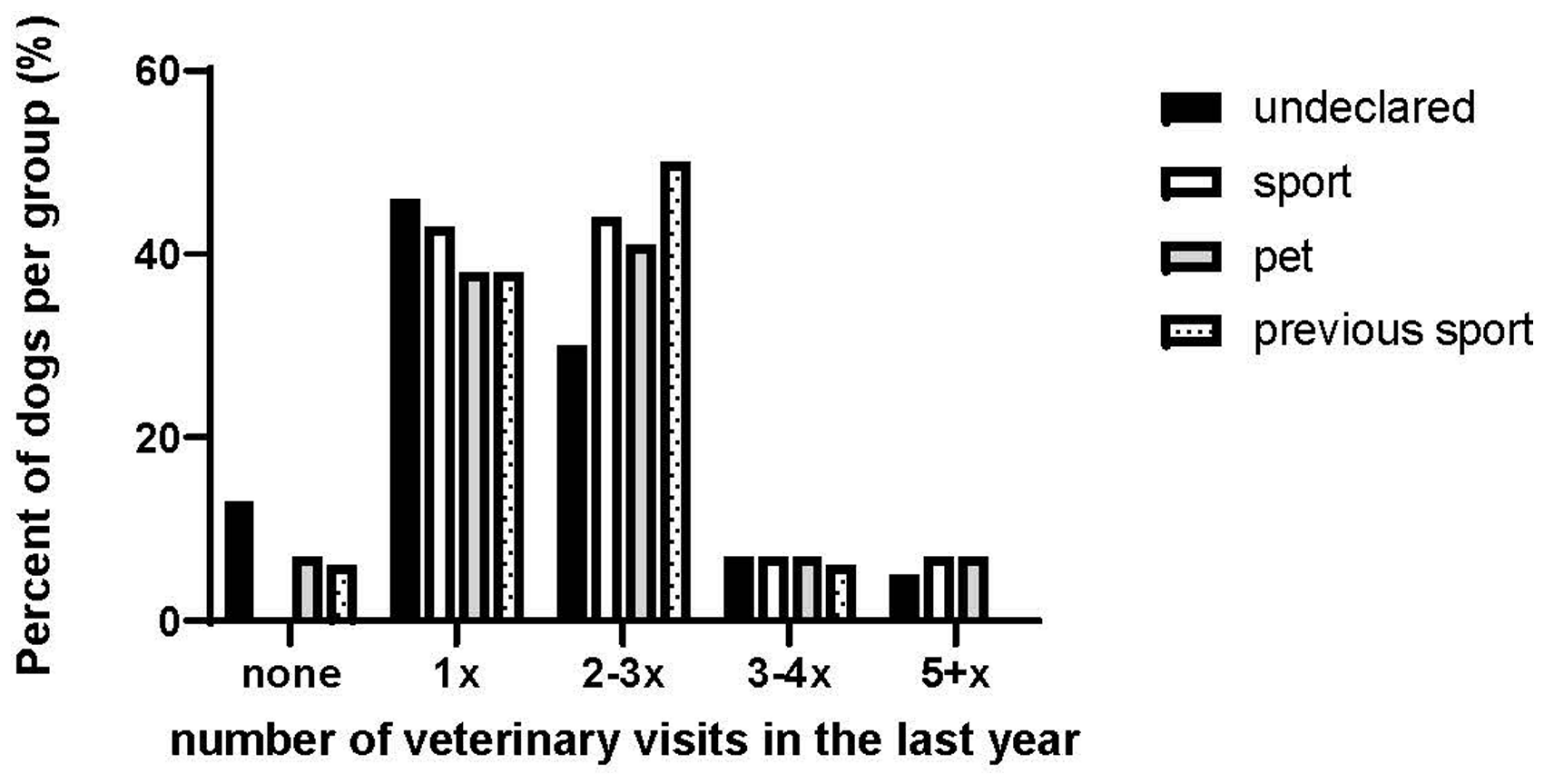
The number of veterinary visits per group.

Owners were asked about previous temporary and permanent diagnoses. Most owners reported no previous health problems in their dogs (Undeclared: 87%; Sport: 89%; Pet: 84%; Previous Sport: 72%). Some temporary orthopedic injuries were reported (Undeclared: 9%; Sport: 8%; Pet: 12%; Previous Sport: 11%). Four dogs were reported as having permanent orthopedic injuries, five had other chronic conditions, one had a permanent cardiorespiratory condition. When asked about the number of vet visits last year, most owners reported between 1–3 vet visits (see Figure 4). A small percentage of owners in the Undeclared (13%; *n* = 11/87), Pet (7%, *n* = 4/58) and Previous Sport (6%, *n* = 1/16) groups reported no vet visits in the last year.

### Dog body condition

**Figure 5:**
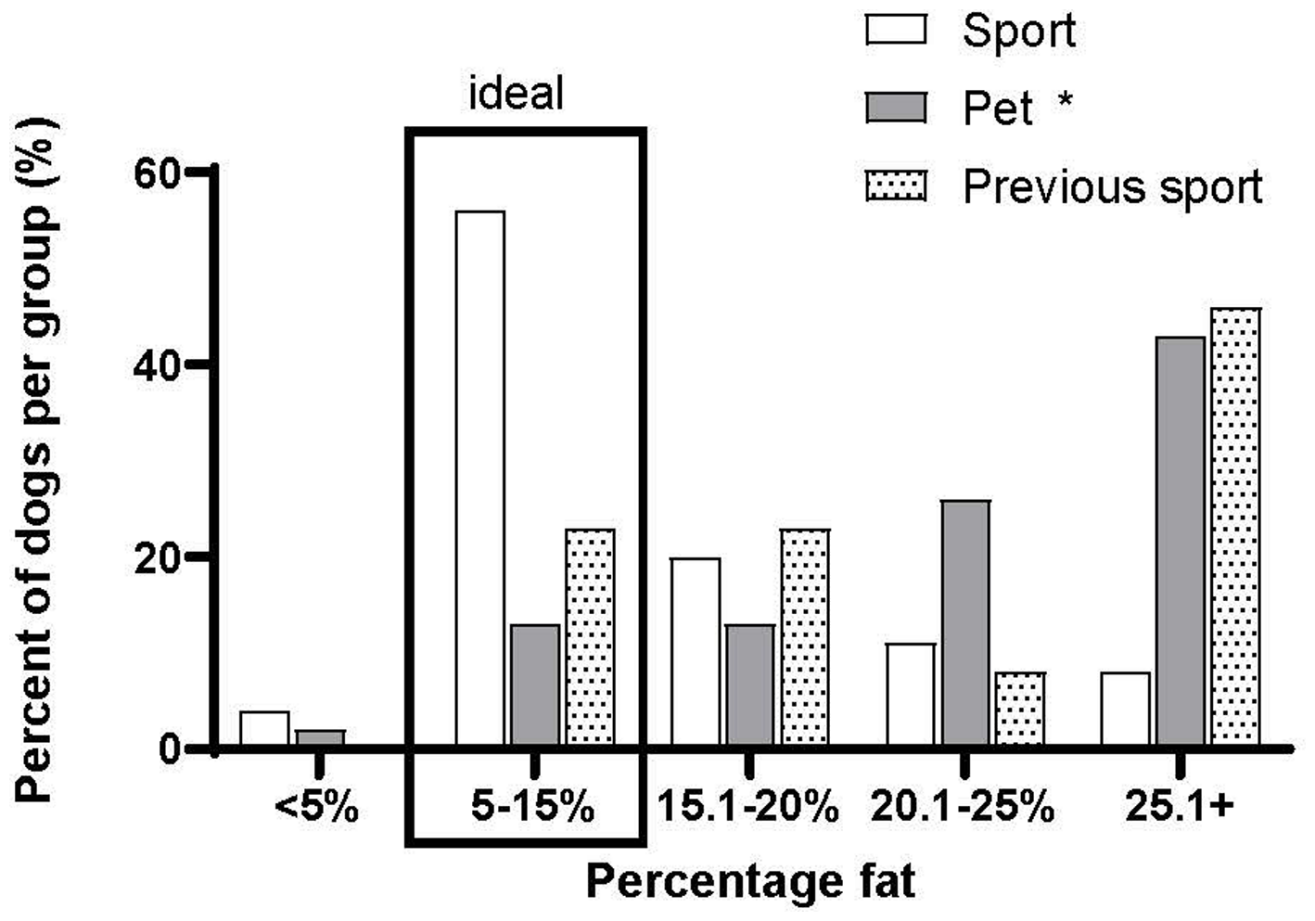
Calculated body fat percentage per group organized by ranges of percent fat. *p < 0.05 mean differences between the Pet group and Sport group.

We asked owners about their dog’s body condition using several methods. We asked if they considered their dog underweight, ideal, overweight or obese. All groups reported, on average, their dogs were in the “ideal” category. We also asked participants to look at a Purina Body condition scale without the weight categories (underweight, ideal etc). Owners in all groups reported a 5 ± 1 score (ideal on Purina scale). Participants also measured their dog’s pelvic circumference and hock to stifle length. In the Undeclared group, only three owners answered these questions, so this group was excluded from the analysis. We calculated percent fat from these measures. Percent fat calculated from owner measurements was significantly different between groups (Sport: 16 ± 10%fat, *n* = 71; Pet: 24 ± 10% fat, *n* = 46; Previous Sport: 23 ± 7% fat, *n* = 13; Sport different from Pet *p* < 0.001; *F* = 21.27). Figure 5 shows the percentage of dogs that fell into percent fat categories. The undeclared group was left out of the analysis because of a large number of missing values. The correlation (Pearson Product Moment) was *r* = 0.25 (*p* < 0.05) for the owner-determined Purina Body condition score and calculated percent fat. Overall, 55% of the Sports dog owners correctly identified their dogs body condition (categories vs. % fat), but only 13% of the pet dog owners correctly identified the body condition of their dog. We investigated the possibility that spay/neuter status played a role in the pet versus sport differences in body condition. The calculated percent fat for intact males was 15 ± 7% fat, neutered males were 18 ± 9% fat, intact females were 23 ± 16% fat, neutered females were 22 ± 10% fat. There were no significant differences between intact and spay/neutered dogs for percent fat and, when sex was taken into account, there were also no significant differences (*p* = 0.15, *F* = 1.92).

### Dog exercise

**Figure 6:**
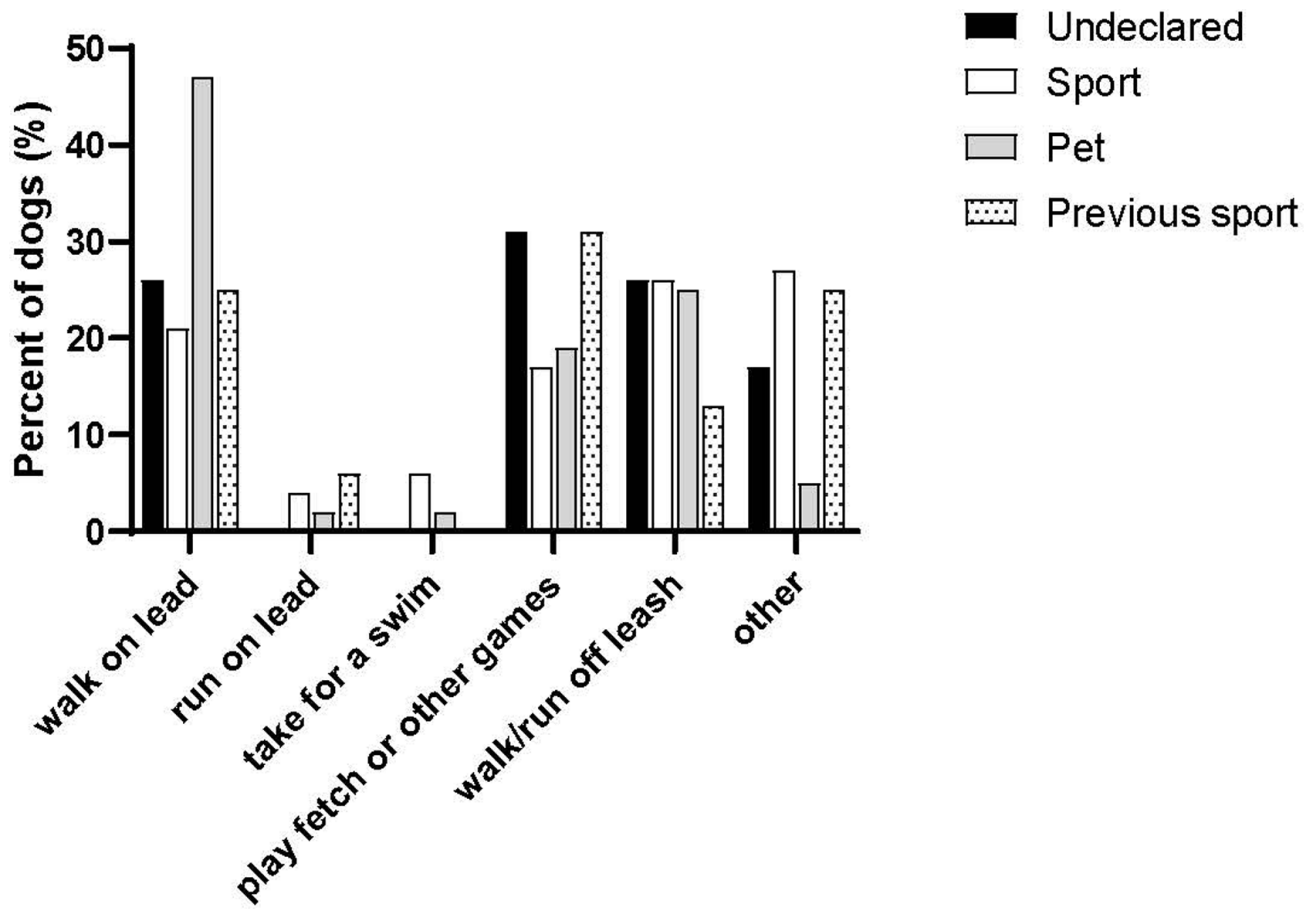
Reported physical activities performed with the dog by group.

Days per week an owner exercised their dog was similar in the Undeclared (9 ± 6 times/week, *n* = 30/120), Sport (7 ± 4 times/week, *n* = 72/90), Pet groups (8 ± 6 times/week, *n* = 58/58) and Previous Sport groups (8 ± 5 times/week, *n* = 11/16) (*F* = 0.72, *p* = 0.54). The types of exercise by group are summarized in Figure 6. The main type of exercise was walking on a lead. Playing fetch and walking or running off leash was also similar between the groups. The Sport group had a large (27%)” other” component that related to their sport activities. The most common length of time per session was 15-30 min for the Sport (42%; *n* = 90) and Undeclared groups (37%, *n* = 35). The most common length of time for the Pet group was 30-45 min (37%; *n* = 57). There was not a significant difference for time reported for activities without the owner (Undeclared: 5 ± 7; Sport: 4 ± 8; Pet: 3 ± 7; Previous Sport: 2 ± 2 h) (*F* = 0.89, *p* = 0.45). The Sport group (52%) spent 1-3 h sport training per week and 0–3 h (77%) on conditioning/foundation exercises. Using the DAWGS survey, the overall positivity about dog walking (Sport: 59 ± 8; Pet: 60 ± 7; Previous Sport: 55 ± 14; max 70; *F* = 2.2, *p* = 0.11) and positivity about the outcomes from dog walking (Sport: 55 ± 11; Pet: 56 ± 9; Previous Sport: 53 ± 13; max 70; *F* = 0.71, *p* = 0.50) was similar between groups. The Undeclared group was not analyzed because these questions were not answered.

### Dog feeding

**Figure 7:**
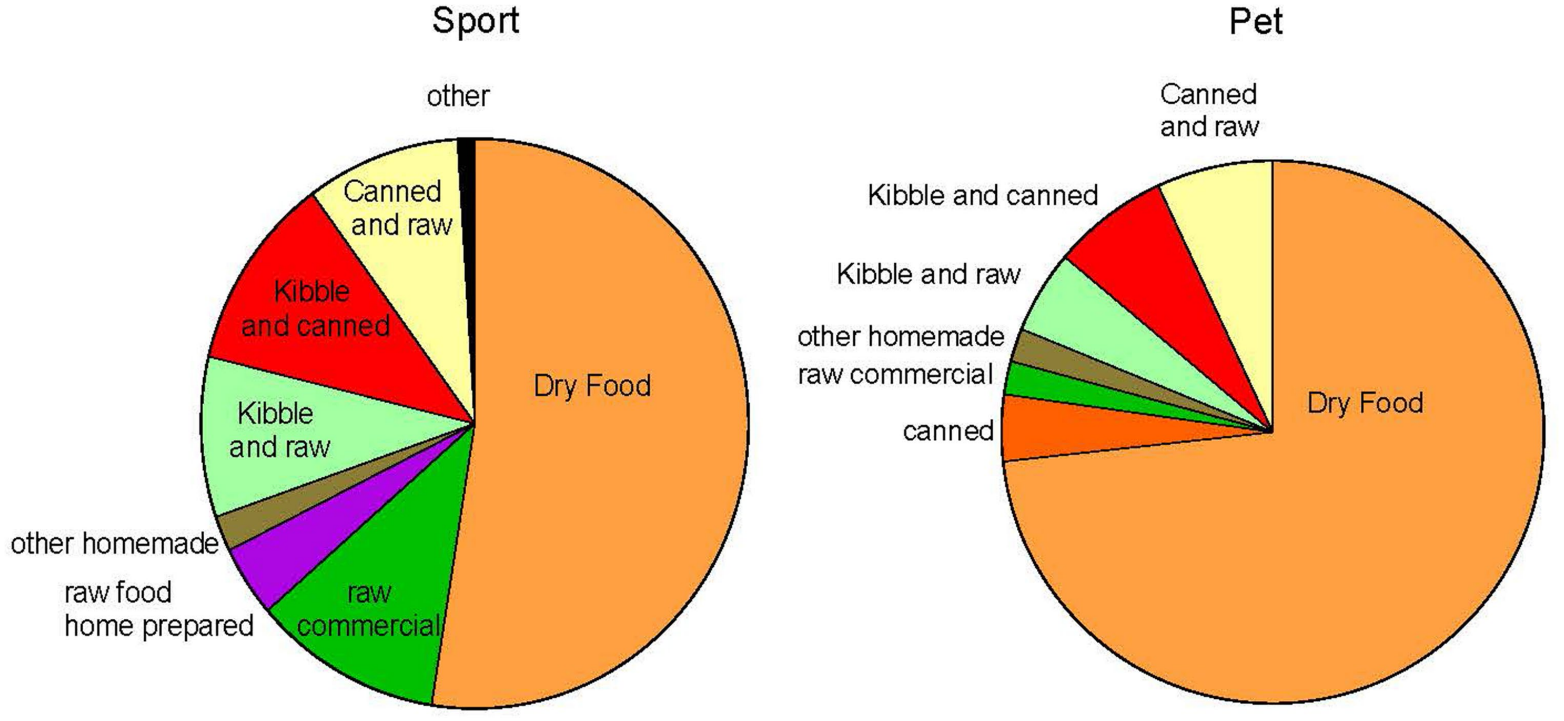
Pictoral description of reported feeding methods in the Pet and the Sport group.

Most owners fed dry kibble food (Undeclared: 60%; Sport: 52%; Pet: 74%; Previous Sport: 63%). The most popular brand was Purina (Undeclared: 22%; Sport: 19%; Pet: 29%; Previous Sport: 31%). Feeding a raw diet was more common in the Sport group (Undeclared: 10%; Sport: 15%; Pet: 2%; Previous Sport: 0%). Figure 7 is a summary of diets used by the Sport and Pet groups. Sport owners (kibble fed) reported feeding dogs a range of 497 ± 196 kcal (small dogs) to 1,109 ± 465 kcal (large dogs) and pet owners (kibble fed) reported 413 ± 158 kcal (small dogs) to 1,133 ± 434 kcal (large dogs) (Undeclared: 447 to 926; Previous Sport: 373 to 1,249 kcal small to large). The correlation between dog size and Kcal was R = 0.58 (*n* = 105; *p* < 0.05). Most owners fed the dog 2x/day (Undeclared: 77%; Sport: 86%; Pet: 60%; Previous Sport: 71%). Free feeding was more common in the Pet group (undeclared: 8%; pet:18%; sport: 1%; previous sport: 0%). Owners reported feeding treats as snacks 1-2x/day (Undeclared: 47%; Sport: 41%, Pet: 47%; Previous Sport: 31%). Undeclared (18%), Sport owners (12%) and Previous Sport (19%) reported feeding no treats as snacks compared to Pet owners (3%). The size of the treat also was smaller in the Undeclared (pinky nail to thumbnail sized; 68%) and Sport dogs (60%) than in Pet dogs (28%) and Previous Sport dogs (25%). Pet owners reported feeding larger sized treats (bigger than thumb to larger than palm; 34%) compared to Sport owners (12%)(Undeclared: 0%, Previous Sport: 13%) (*p* = 0.002, *F* = 6.426).

### Dog related expenses

We looked at total dog costs per month, undeclared ($419 ± 505) and Sport groups ($321 ± 167) spent more than the Pet ($123 ± 82; *p* < 0.05 different from Undeclared and Sport; *F* = 12.26, *p* < 0.01).

## Discussion

A strength of this study was we were able to sample dog owners around the US and a small number in Canada. We also included actual measurements of the dog so body fatness could be directly assessed. The most important finding was that most owners perceived their dogs to be in an “ideal” body condition, but measurements revealed that over 50% of the dogs were over fat. In general, pet dogs were considerably more over-fat compared to sports dogs. Dog exercise was consistent across groups and suggested that owners walked with their dogs most days of the week for 15 to 45 min. The most common type of food fed was dry kibble for many dogs and the calories that the owners reported feeding was consistent across groups. The number of treats fed was consistent across groups, but the pet group reported feeding a larger size of treat. The Sport group spent more money on their dogs including food, toys, leashes and training. Despite considerable commitment by the owners to maintain their dog’s health and well-being, many of the dogs were over fat and owner’s assessment of their dog’s body condition was very flawed in the sport and pet groups.

### Dog demographics and health

The sample represented around 38 states and two regions of Canada. The largest representation was on the east coast of the US. The dogs were on average middle aged, spayed/neutered and medium sized. We did see an effect of age among groups, with the pet group having a slightly higher age than the other groups. However, this is a statistical difference without meaning because the means were 5 years versus 6 years. We had a large representation of breeds with no one breed being predominant in any owner category. Owners reported that their dogs were generally healthy and visited the vet at least 1–3 times per year. This finding was consistent with previous research suggesting that at least 40% of pet owners visited the veterinarian at least once per year (7)(AVMA 2017–2018). We reported that 7% of Pet owners reported no contact with the veterinarian. This was higher than reported by Bir et al. (7), but lower than reported by the AVMA US Pet Ownership Statistics 2017–2018.

### Dog body condition

One of the most concerning findings in this study was that 55% of the Sport dog owners correctly identified their dogs body condition, but only 13% of the pet dog owners correctly identified the body condition of their dog. The percent correctly identifying body condition in the Sports dog owners was consistent with our previous work (4) and the work of others (8–10). Thirteen percent correct in the Pet owner group was very low and of considerable concern because many of these dogs were significantly over fat according to the owner reported measurements. Our hopes that owners would be able to use the Purina scale descriptions effectively was not realized, since most owners reported their dogs had” ideal” body condition. The finding that good descriptions of body condition do not overcome owners preconceived notions about their dog’s body condition was consistent with Eastland-Jones et al. (9). It appears that more education is required regarding the use of the Purina scale or other measurements to optimize assessment of body condition by pet owners. It is unlikely that an owner would implement a change in feeding or exercise if they could not identify their dog as overfat.

### Dog exercise

In general, exercise was very comparable between Pet and Sport dogs. The most common exercise in all groups was walking and most owners reported walking their dogs every day for 15–45 min per session. This is consistent with the recommendations for physical activity for dogs and humans (11). Our participants reported walking with their dog more than participants in Christian et al. (12) and a similar amount compared to Hoerster et al. (13) and Banton et al. (14), but less than Kinsman et al. (15). Based on a write in question from our previous study (4), we asked several questions about activities that the dog did without the owner, but found no difference for time reported for activities without the owner. The Sport group spent 1–3 h sport training per week and 0–3 h on conditioning/foundation exercises. This emphasizes that sport dogs get a reasonable amount of exercise that is specific to their sport, in addition to traditional activities like walking (16). Using the DAWGS survey, the overall positivity about dog walking and positivity about the outcomes from dog walking was similar among groups and consistent with other studies (5, 17, 18).

### Dog feeding

Most owners fed dry kibble food and the most popular brand was Purina. Feeding a raw diet was more common in the Sport group. Dinallo et al. (19) reported that 61% of agility competitors fed commercial dry kibble and 25% fed raw or other homemade food. This was similar in flyball competitors (33% fed raw; 61% fed kibble) (20). Sport and Pet owners reported feeding dogs a range of approximately 400 to 1,130 kilocalories (Kcal). There was a moderate correlation between dog size and kilocalories fed. This correlation is good because it suggests at least some adherence to the idea that a smaller dog requires fewer kilocalories. Most owners fed the dog two times per day. This practice is consistent with good feeding practices from studies comparing obese to non-obese dogs (8, 9, 21, 22). Free feeding was more common in the Pet group but practiced minimally in the Sport group. Feeding of treats was an area that was different between Sport and Pet owners. We specifically asked about treats as snacks versus treats used for training. Most owners reported feeding treats as snacks one to two times per day, but a reasonable percent of Sports owners said they did not feed treats as snacks. We asked about the size of the treat using a person’s hand as a reference. The majority of Sports dog owners reported a treat of a pinky nail to thumbnail sized. However, Pet owners reported feeding treats that were bigger than thumb to larger than palm. A popular large sized treat can add 125 kilocalories or more to a dog’s daily intake. It is well understood that snack feeding can contribute to obesity in dogs (8, 21, 23, 24). Therefore, it is important to counsel owners to curb this practice of using treats as snacks for their dog.

### Dog expenses

Overall, the Sport group spent more than twice as much in total dog costs per month compared to the Pet group. This is supported by the idea that owners participating in dog sports activities have a “culture of commitment” that often involves considerable sacrifice of money and time for the dog (25). However, spending money on the dog’s comfort and well-being is not exclusive to sport and often relates to an owner’s perceived level of dog-human companionship (26, 27).

### Limitations

The anthropometric measurements in this study were taken by the owner rather than a researcher due to the questionnaire based nature of the study. It is possible that owners made measurement errors in determining the pelvic circumference and hock to stifle length. However, it is unlikely that the error was subjectively biased because the owner was not aware of the results of the percent fat calculation from the measurements. It is not common to report the training of people taking anthropometric measurements in humans (28) and dogs (6, 29, 30) because it is generally considered a fairly simple measure.

Another limitation was the assessment of intensity of exercise in the dogs. We were unable to assess relative intensity (low, moderate, vigorous) from the questionnaire information. In humans, exercise intensity is well documented for a variety of activities, but, in dogs, this literature dos not exist and is likely complicated by the large variation in dog size in different breeds. In the future, the use of wearables for dogs may improve the estimation of exercise intensity.

### Conclusion

Despite considerable time and financial commitment to their dogs, owners in the Sport and Pet groups did a poor job at correctly assessing their dogs body condition. This is a concerning finding since it is unlikely that a person that perceives their dogs body condition to be “ideal” will implement changes in feeding or exercise. The owners in this study generally did a creditable job in regularly exercising their dogs and most fed their dogs using accepted practices to maintain body condition. However, the size of treats was larger in the most over fat dogs. Better understanding of correct feeding for the level of physical activity is still a critical issue in controlling obesity in pet dogs.

## Data availability statement

The datasets presented in this study can be found in online repositories. The names of the repository/repositories and accession number(s) can be found at: https://hak0006.wixsite.com/vascularphyslab/data.

## Ethics statement

The studies involving humans were approved by Auburn University Institutional Review Board. The studies were conducted in accordance with the local legislation and institutional requirements. Written informed consent for participation in this study was provided by the participants' legal guardians/next of kin. The animal studies were approved by Auburn University Institutional Review Board. The studies were conducted in accordance with the local legislation and institutional requirements. Written informed consent was obtained from the owners for the participation of their animals in this study. Written informed consent was obtained from the individual(s) for the publication of any potentially identifiable images or data included in this article.

## Author contributions

HK participated in all parts of this project. RJ contributed to the data collection and editing the manuscript. All authors contributed to the article and approved the submitted version.
